# The *FTO *(fat mass and obesity associated) gene codes for a novel member of the non-heme dioxygenase superfamily

**DOI:** 10.1186/1471-2091-8-23

**Published:** 2007-11-08

**Authors:** Luis Sanchez-Pulido, Miguel A Andrade-Navarro

**Affiliations:** 1 Centro Nacional de Biotecnologia, CSIC, Madrid, Spain; 2 Molecular Medicine, Ottawa Health Research Institute, Ottawa, Canada; 3 Faculty of Medicine, University of Ottawa, Ottawa, Canada; 4 Max Delbrück Center for Molecular Medicine, Berlin, Germany

## Abstract

**Background:**

Genetic variants in the *FTO *(fat mass and obesity associated) gene have been associated with an increased risk of obesity. However, the function of its protein product has not been experimentally studied and previously reported sequence similarity analyses suggested the absence of homologs in existing protein databases. Here, we present the first detailed computational analysis of the sequence and predicted structure of the protein encoded by *FTO*.

**Results:**

We performed a sequence similarity search using the human FTO protein as query and then generated a profile using the multiple sequence alignment of the homologous sequences. Profile-to-sequence and profile-to-profile based comparisons identified remote homologs of the non-heme dioxygenase family.

**Conclusion:**

Our analysis suggests that human FTO is a member of the non-heme dioxygenase (Fe(II)- and 2-oxoglutarate-dependent dioxygenases) superfamily. Amino acid conservation patterns support this hypothesis and indicate that both 2-oxoglutarate and iron should be important for FTO function. This computational prediction of the function of FTO should suggest further steps for its experimental characterization and help to formulate hypothesis about the mechanisms by which it relates to obesity in humans.

## Background

Two recent reports [1,2] characterized the strong association of a number of single nucleotide polymorphisms (SNPs) in intron 1 of the human *FTO *gene with an increased risk of obesity, characterized by an increase in body max index due to fat mass rather than lean mass that is seen in children as early as age seven [2].

However, the mechanisms by which this genetic variability relates to obesity remain obscure. These publications indicate that the function of *FTO *is unknown [2] and that its protein has no identified structural domain or link to other proteins that could be used to predict its function [1]. Knowledge of the function of *FTO *is crucial to guide the search for a mechanism relating this gene to obesity.

Here we report evidence obtained by computational analysis indicating that the protein coded by *FTO *is a member of the non-heme dioxygenase (Fe(II)- and 2-oxoglutarate-dependent dioxygenases) superfamily.

## Results and Discussion

**Figure 1:**
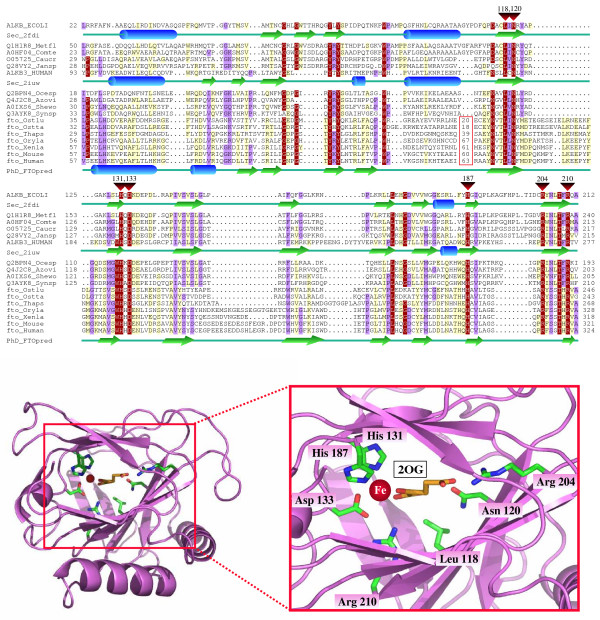
**Computational analysis of the FTO family**. **Top**. Multiple sequence alignment of the FTO family with known members of the non-heme dioxygenase superfamily. Red triangles above the alignment mark the conserved residues involved in iron and 2-oxoglutarate (2OG) binding with numbers indicating their position in AlkB. The numbers within the red box represent sequence insertions that we did not include in the alignment. X-Ray determined secondary structure of AlkB (PDB code 2fdi) [5] and hABH3 (PDB code 2iuw) [4] are shown below their sequences. PHD secondary structure prediction [26] for the FTO family is included below the human FTO sequence. The alignment was produced using a combination of T-COFFEE [17] and profile-to-profile alignment [24], using the structure-based superposition hABH3/AlkB alignment [28] as reference. Finally, the alignment was slightly refined manually. It was represented with the program Belvu [18] with a coloring scheme indicating average BLOSUM62 score (correlated to amino acid conservation) in each alignment column: dark red (greater than 3), violet (between 3 and 1) and light yellow (between 1 and 0.3). The sequences are named with their SwissProt or SpTrembl identifiers. Species abbreviations: Azovi, *Azotobacter vinelandii*; Caucr, *Caulobacter crescentus*; Comte, *Comamonas testosteroni*; Ecoli, *Escherichia coli*; Human, *Homo sapiens*; Jansp, *Jannaschia sp.*; Metfl, *Methylobacillus flagellatus*; Mouse, *Mus musculus*; Ocesp, *Oceanospirillum sp.*; Oryla, *Oryzia latipes*; Ostlu, *Ostreococcus lucimarinus*; Ostta, *Ostreococcus tauri*; Shewo, *Shewanella woodyi*; Synsp, *Synechococcus sp.*; Thaps, *Thalassiosira pseudonana*; Xenla, *Xenopus laevis*. Additional details about some lanes and FTO close homologous sequences can be found in Table 1. Complementary information, sequences, and alignments are accessible at [29]. **Bottom**. Structure of AlkB (PDB code 2fdi) indicating with sticks the invariant side chains in non-heme dioxygenases which are also conserved in the FTO family.

In the course of the computational characterization of the FTO family (see Methods) we identified sequences homologous to human FTO in different eukaryote groups including vertebrates (from fish to mammals), green algae (*Ostreococcus*) and diatoms (*Phaeodactylum *and *Thalassiosira*) (see Figure 1 and Table 1).

**Table 1:** Additional details for lanes in Figure 1 and FTO close homologous sequences in Figures 1 and 2.

**Identifier**	**Obtained from**	**Species**	**Representing**
ALKB_ECOLI	SwissProt	*Escherichia coli*	Alpha-ketoglutarate-dependent dioxygenase AlkB
Sec_2fdi	2D structure		
ALKB3_HUMAN	SwissProt	*Homo Sapiens*	hABH3 (human AlkBHomolog 3)
Sec_2iuw	2D structure		
fto_Ostlu	gi:145352974 & FGENESH+^1^	*Ostreococcus lucimarinus*	Homolog to FTO
fto_Ostta	gi:116060758 & FGENESH+^1^	*Ostreococcus tauri*	Homolog to FTO
AACY020075623	AACY020075623 & FGENESH+^1^	Marine metagenome	Homolog to FTO (not shown in Fig. 1)
fto_Thaps	JGI database & FGENESH+^1^	*Thalassiosira pseudonana*	Homolog to FTO
fto_Phatr	JGI database & FGENESH+^1^	*Phaeodactylum tricornutum*	Homolog to FTO (not shown in Fig. 1)
fto_Oryla	Ensembl^1^	*Oryzia latipes*	Homolog to FTO
fto_Xenla	Sptrembl: Q68F54^1^	*Xenopus laevis*	Homolog to FTO
fto_Xentr	UniRef100: UPI0000509E0B	*Xenopus tropicalis*	Homolog to FTO
fto_Bosta	UniRef100: A5D798	*Bos taurus*	Homolog to FTO
fto_Canfa	JGI database & FGENESH+^1^	*Canis familiaris*	Homolog to FTO
fto_Ratno	UniRef100: Q2A121	*Rattus norvegicus*	Homolog to FTO
fto_Mouse	SwissProt: FATSO_MOUSE	*Mus musculus*	Homolog to FTO
fto_Human	SwissProt: FATSO_HUMAN^1^	*Homo sapiens*	FTO
PhD_FTOpred	2D Structure^2^		

^1^Manually assembled ESTs from NCBI [31] and homologous sequences were used in order to produce accurate gene predictions supported by homology information.

^2^Secondary structure prediction using PHD [26].

Using sequence profiles of the N-terminal conserved region of the FTO family (corresponding to the human FTO sequence amino acid positions 57–324) members of the non-heme dioxygenase family were identified. Additionally, the secondary structure predictions of the FTO family showed high similarity with the known structures of AlkB, a member of the non-heme dioxygenase family [3-5]. We were not able to find significant homology in the C-terminal of the FTO family to other genes.

To investigate if fold recognition analysis would generate supporting results, we submitted the FTO N-terminal region as a query to an independent fold assignment system based on profile-profile comparisons (see Methods). The profiles generated for the human and *E. coli *AlkB proteins (PDB entries 2iuw and 2fdi) matched the FTO N-terminal region with an E-Value of 3.2 × 10^-21 ^and 3.1 × 10^-12^, respectively (estimated error rate < 3%) despite their low level of sequence identity to the human FTO protein (approximately 17%). The next match corresponded to the hypothetical protein TM0957 from *Thermotoga maritima*, however it was considered unreliable given its short length (28 amino acids) and high E-value (0.02).

Given the E-values of the HMMer searches, the reliability of secondary structure predictions, and the fold assignment results, we are confident that the proteins of the FTO family (including the protein coded by the *FTO *human gene) are members of the non-heme dioxygenase superfamily.

Proteins of this superfamily catalyze different oxidative reactions on multiple substrates producing varied biological effects [6] and are characterized by a number of conserved amino acids involved in the binding of iron and 2-oxoglutarate (as a cofactor and co-substrate, respectively). We found these amino acids in human FTO and in its homologs (see Figure 1 and Table 1), suggesting that 2-oxoglutarate and iron are essential for the normal function of the FTO protein.

The FTO family is not a unique case as other families of the non-heme dioxygenase superfamily are also very divergent and their detection required non-trivial computational analysis [3]. Due to the divergence of the FTO family from already known non-heme dioxygenases, we were unable to predict the target of the family's catalytic action.

The ubiquitous expression of FTO throughout many human tissues [1] indicates that it has an important function. The phylogenetic distribution of FTO homologs (consistently present in organisms from fish to mammals) suggests that this gene appeared during the evolution of vertebrates. Intriguingly, FTO homologs can be found in green algae *Ostreococcus *and diatoms, whereas they are apparently absent in insects, worms and fungi (see Figure 1 and Table 1). The most parsimonious explanation of this fact is the existence of independent events of horizontal gene transfer from vertebrates to protists. Horizontal gene transfer has previously been related to the evolution of several eukaryotic regulatory systems that function in development, differentiation and apoptosis [7]. Concisely, horizontal transfer of FTO indicates that the FTO protein has a function that confers a selective advantage but that it is not indispensable, which agrees with a possible regulatory role.

For comparison, the hypoxia-inducible factor (HIF), a known member of the non-heme dioxygenase family, also has a wide phylogenetic distribution (from worms to mammals) and is ubiquitously expressed in all human tissues. HIF acts as a sensor of oxygen level and affects the expression of over one hundred genes [8]. This molecule performs its activity by shuttling between the cytoplasm in normoxic conditions and the nucleus in hypoxic conditions [9].

To investigate if FTO could be acting in a similar manner, we studied its sequence using an algorithm for prediction of protein cellular localization (WolfPSORT; [10]). The results suggested with similar scores a cytoplasmic and a nuclear-cytoplasmic localization for this protein. This is consistent with human FTO's possible function as a metabolic sensor and nuclear effector.

**Figure 2:**
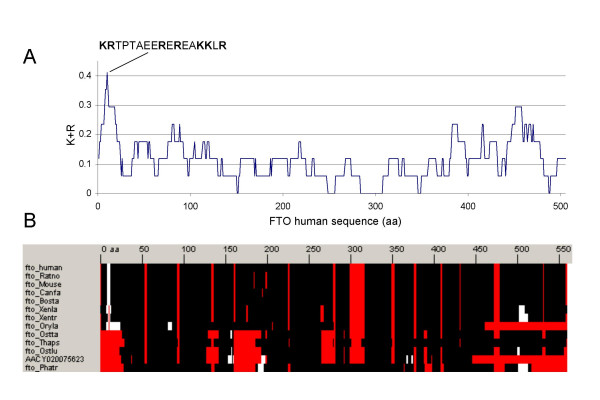
**Analysis of K/R rich regions in the FTO family**. (A) Plot of the percentage of K/R residues in a window of 17 amino acids of human FTO. The sequence fragment indicated from position 2 to 18 includes the maximum (7/17 at position 10). (B) Representation of the multiple (full) sequence alignment of the FTO family. White indicates regions with more than 30% of K+R residues in a window of 17 amino acids along the aligned sequences and red represents gaps in the alignment. The N-terminal region around the predicted bipartite NLS signal of human FTO stands as the only K/R-rich region conserved in fish as well as mammalian sequences. Both plots were generated using the BiasViz java tool [30].

The FTO human gene product has a predicted molecular mass of 50 KDa. With that mass it would need a Nuclear Localization Signal (NLS) [11] in order to act in the nucleus. Analysis of FTO's sequence using an algorithm that includes the prediction of NLS (PSORTII; [10]) suggested a 17 amino acid long bipartite NLS from positions 2 to 18 (Figure 2A) noted previously [12] but not experimentally verified. Further analysis of the family indicated that this region stands as a K/R rich region in comparison to the rest of the sequence, and that it is located in an N-terminal extension that is conserved in close human homologs from fish to mammals but not in the other FTO homologues we found in algae or diatomea.

In light of these computational results we hypothesize that FTO is a sensor of the cell's metabolic state and when dysfunctional can result in an obese phenotype. We identify the N-terminal of human FTO as having a high likelihood of determining its cellular localization, which could be verified by mutational analysis.

## Conclusion

Here we have provided valuable information about FTO by indicating its possible catalytic function, and we have pointed to the amino acids involved in cofactor (Fe) and co-substrate (2-oxoglutarate) binding in human FTO as well as in its homologous proteins in other organisms, which could be used as models for the study of the human disease. This insight should help to guide experiments to clarify the mechanisms by which *FTO *relates to obesity and to accelerate the discovery of novel molecular therapies for this condition.

## Methods

We first performed BLAST sequence similarity searches [13] using the human FTO protein as query against different sequence database resources: NCBI [14], ENSEMBL [15] and JGI [16]. Multiple sequence alignments of protein sequences homologous to human FTO were generated with the program T-Coffee [17] using default parameters, slightly refined manually and visualized with the Belvu program (Figure 1. Top) [18].

Profiles of the alignment as global hidden Markov models (HMMs) were generated using HMMer [19]. Profile-based sequence searches were performed against the Uniref50 and Uniref90 protein sequence databases [20] using HMMsearch [21]. We used NAIL [22] to view and analyze the HMMsearch results, which provided a formatted view with hyperlinks to related web resources and coloring related to taxonomic information, thus facilitating the interpretation of the results.

Fold recognition analyses were performed using profile-to-profile comparisons of the HMM profile of the FTO family to profiles generated for each sequence of known structure with its homologues (HHpred server; [23,24]). The significance of sequence-to-sequence, profile-to-sequence, and profile-to-profile matches were evaluated in terms of an E-value, which is an estimation of the probability of finding a better match by chance. Secondary structure predictions were performed using the PredictProtein Server [25,26]. AlkB active center illustrations (Figure 1. Bottom) were generated with Pymol [27].

## Abbreviations

2OG (2-oxoglutarate)

AlkB (Alkylated DNA repair protein)

ESTs (Expressed sequence tags)

FGENESH (Find Genes using HMM)

FTO (fat mass and obesity associated)

HIF (Hypoxia-inducible factor)

HMMs (Hidden Markov Models)

JGI (Joint Genome Institute)

NBCI (National Center for Biotechnology Information)

NLS (Nuclear Localization Signal)

SNPs (Single nucleotide polymorphisms)

## Authors' contributions

LSP carried out the initial sequence and structural analysis of the domain. LSP and MAA interpreted the data and prepared the manuscript. All authors read and approved the final manuscript.
